# Visual field examinations using different strategies in Asian patients taking hydroxychloroquine

**DOI:** 10.1038/s41598-022-19048-0

**Published:** 2022-08-30

**Authors:** Ko Eun Kim, So Jung Ryu, Young Hwan Kim, Yuchan Seo, Seong Joon Ahn

**Affiliations:** 1 Department of Ophthalmology, Asan Medical Center, Ulsan University School of Medicine, Seoul, Republic of Korea; 2 Department of Ophthalmology, Hanyang University Hospital, Hanyang University College of Medicine, 222-1 Wangsimni-ro, Seongdong-gu, Seoul, 04763 Republic of Korea; 3 Hanyang University College of Medicine, Seoul, Republic of Korea

**Keywords:** Eye diseases, Diagnosis

## Abstract

In this study, we investigated the patterns of visual field (VF) defects and the diagnostic abilities of VF tests using different strategies in Asian patients with hydroxychloroquine retinopathy. Patients screened for hydroxychloroquine retinopathy using optical coherence tomography, fundus autofluorescence, VF, and/or multifocal electroretinography were included. The VF was performed using the Humphrey 30-2 and/or 10-2 strategy, and 2,107 eyes of 1,078 patients with reliable results, including 136 eyes of 68 patients with hydroxychloroquine retinopathy, were analyzed. The characteristics of VF findings were evaluated and the sensitivity and specificity were compared between the 30-2 and 10-2 tests in subgroups of retinopathy severity and pattern. The most common VF defect pattern was partial- or full-ring scotoma in both the 10-2 and 30-2 tests. Among the eyes with hydroxychloroquine retinopathy that underwent both tests, 14.2% showed a disparity between the two tests, almost all at the early stage. In overall and early pericentral retinopathy, the sensitivity of the 30-2 test was significantly higher than that of the 10-2 test (95.7% vs. 77.1% and 90.6% vs. 53.1%, respectively; *P* < 0.05). However, the specificity of the 10-2 test was significantly higher than that of the 30-2 test (89.6% vs. 84.8%, *P* < 0.001). Therefore, the pattern of retinopathy should be carefully considered when choosing a VF strategy for better detection of hydroxychloroquine retinopathy.

## Introduction

Hydroxychloroquine is widely used for the treatment of rheumatic and dermatologic diseases such as systemic lupus erythematosus and rheumatoid arthritis. Despite its therapeutic benefits, retinal toxicity is of serious concern, and not only for ophthalmologists but also other physicians^1,2^. The most recent American Academy of Ophthalmology (AAO) recommendations published in 2016 highlighted the importance of screening for hydroxychloroquine retinopathy using four tests: spectral-domain optical coherence tomography (SD-OCT), automated visual field (VF) test, multifocal electroretinography (mfERG), and fundus autofluorescence (FAF). According to the guidelines, at least one objective test result is necessary to confirm a subjective test abnormality, unless the retinopathy is obvious and advanced^3^.

Standard automated perimetry (SAP) is considered to be one of the key screening tests, as it provides important evidence on subjective, functional deterioration caused by the retinal toxicity of hydroxychloroquine^3^. There is compelling evidence that the ring scotoma correspondent with parafoveal retinal damage is prevalent in patients with hydroxychloroquine retinopathy^4,5^. Although 10-2 Humphrey VF test has been recommended for screening^5–7^, VF results may vary depending on the location (pattern) of hydroxychloroquine retinopathy and also the test strategy^3,8^. For example, Lee et al. reported a case with pericentral hydroxychloroquine retinopathy showing a localized scotoma found on the 30-2 test, but not on the 10-2 test^8^. The pattern of retinopathy, either parafoveal or pericentral, is reported to be dependent on ethnicity; for example, pericentral retinopathy alone was seen in 50% of Asian patients, but only in approximately 2%, 5%, and 21% of white, black, and Hispanic patients, respectively^9^. In this regard, the most recent AAO guidelines revised the recommended VF protocols for Asians, suggesting wider tests (e.g., 24-2, 30-2)^3^. However, data on the diagnostic performance of VFs with different testing strategies in patients with different patterns and severity of retinopathy remains insufficient.

Consequently, we analyzed VF findings in a large number of screened patients, including those with diverse patterns and severities of hydroxychloroquine retinopathy. VF findings in eyes with early retinopathy are relatively unknown, but important for early detection; these were analyzed separately. Finally, we aimed to determine which VF testing strategy is suitable for each pattern of retinopathy by evaluating the diagnostic abilities of the 10-2 and 30-2 VF tests according to the patterns and severities of retinopathy.

## Methods

### Patients

We retrospectively reviewed the medical records of a cohort of 1,221 consecutive Asian patients with a history of taking hydroxychloroquine who had undergone ophthalmic examinations to screen for hydroxychloroquine retinopathy at Hanyang University Hospital between January 2016 and December 2020. A total of 301 eyes of 161 patients were excluded due to unavailable or unreliable VF tests. Further, patients with combined macular diseases (e.g., epiretinal membrane, age-related macular degeneration, central serous chorioretinopathy, macular edema associated with diabetic retinopathy, or retinal vein occlusion), glaucomatous optic neuropathy, other optic disc anomalies or neuropathies (e.g., ischemic optic neuropathy), or neurologic/systemic diseases affecting the VF (e.g., pituitary tumor) were excluded. Finally, 2,107 eyes of 1,078 patients were included in the study (Supplemental Fig. S1). This study adhered to the tenets of the Declaration of Helsinki and was approved by the Institutional Review Board (IRB) of Hanyang University Hospital. The need for informed consent was waived by the IRB due to the retrospective nature of the study.

### Examinations

All of the included patients underwent comprehensive ophthalmic examinations, including slit-lamp examination, measurement of best-corrected visual acuity and refractive error (KW-1500; Kowa, Tokyo, Japan), noncontact tonometry (KT-500 automated tonometer; Kowa), fundus examination using indirect ophthalmoscopy, OCT, FAF, and SAP. Swept-source OCT (DRI-Triton; Topcon Inc., Tokyo, Japan) was performed after pupil dilation. A wide-field 3D macular volume scan that generated a cube of data through a 9 × 12-mm^2^ grid after acquiring a series of 256 B-scans, each of which was composed of 512 A-scans, and line scans (either 12-mm radial scans or 5-line vertical and horizontal raster scans) were used for OCT imaging. Blue-light FAF, obtained using an F-10 confocal scanning laser ophthalmoscope (Nidek, Gamagori, Japan), and/or ultrawide-field FAF (Optos PLC, Dunfermline, UK), also was performed as an additional objective screening test to identify photoreceptor/retinal pigment epithelium (RPE) damage. In patients requiring additional objective evidence for diagnosis of hydroxychloroquine retinopathy, mfERG (Diagnosys LLC, Lowell, MA, USA) was performed according to the International Society for Clinical Electrophysiology of Vision (ISCEV) guidelines^10^.

### VF examination

All of the patients underwent a standardized VF test using the Swedish Interactive Threshold Algorithm 10-2 and/or 30-2 strategy on the HFA II or III (Carl Zeiss Meditec, Dublin, CA, USA). Only reliable VFs defined as fixation loss < 20%, false-positive error < 15%, and false-negative error < 15% were included in the subsequent analysis^11^. All of the VF tests were further examined for the presence of artifacts associated with fatigue, inattention, inappropriate fixation, eyelid or lens rim effect, and any showing such artifacts were excluded from further analyses. In the following comparative analysis of diagnostic performance between the 10-2 and 30-2 tests for detection of hydroxychloroquine retinopathy, only patients who had undergone both tests within a month were included. The two VF strategies were performed randomly for patients who underwent both tests on the same day.

### Diagnosis and classification of hydroxychloroquine retinopathy

For the evaluation of the diagnostic abilities of the VF tests, we used the modified diagnostic criterion (gold standard) for the diagnosis of hydroxychloroquine retinopathy that excludes VF from the most recent AAO guidelines^3^: at least one objective test (FAF or mfERG) abnormality confirming OCT abnormality. As OCT is currently the preferred primary test for diagnosis of hydroxychloroquine retinopathy, OCT abnormality was used as the main requirement for the diagnosis; notably also, ‘OCT abnormality plus additional structural or functional evidence’ is compatible with the most recent Royal College of Ophthalmology guidelines^12^. More specifically, all patients diagnosed with hydroxychloroquine retinopathy show characteristic parafoveal/pericentral photoreceptor defects and/or RPE defects on SD-OCT images^3,13^. Hyper- or hypo-autofluorescence on FAF or decreased amplitude on mfERG in the areas corresponding to outer retinal defects on OCT was used as additional objective evidence for the diagnosis of hydroxychloroquine retinopathy^3,14^.

The severity and pattern of retinopathy were further determined in the eyes with hydroxychloroquine retinopathy. The severity of hydroxychloroquine retinopathy was graded, as in previous reports, as follows: early (patchy, localized photoreceptor defects without RPE involvement on OCT and/or FAF), moderate (photoreceptor damage with partial [> 180°] or full ring on imaging), or severe (combined RPE damage [thinning/attenuation of RPE line on OCT or hypo-autofluorescence on FAF])^3,14^. Depending on the pattern of retinopathy, the eyes with hydroxychloroquine retinopathy were classified as parafoveal (photoreceptor/RPE disruption within 2–6° of fovea), pericentral (outer retinal damage > 6° from fovea), or mixed (both patterns)^3^.

### Evaluation of VF test results

**Figure 1:**
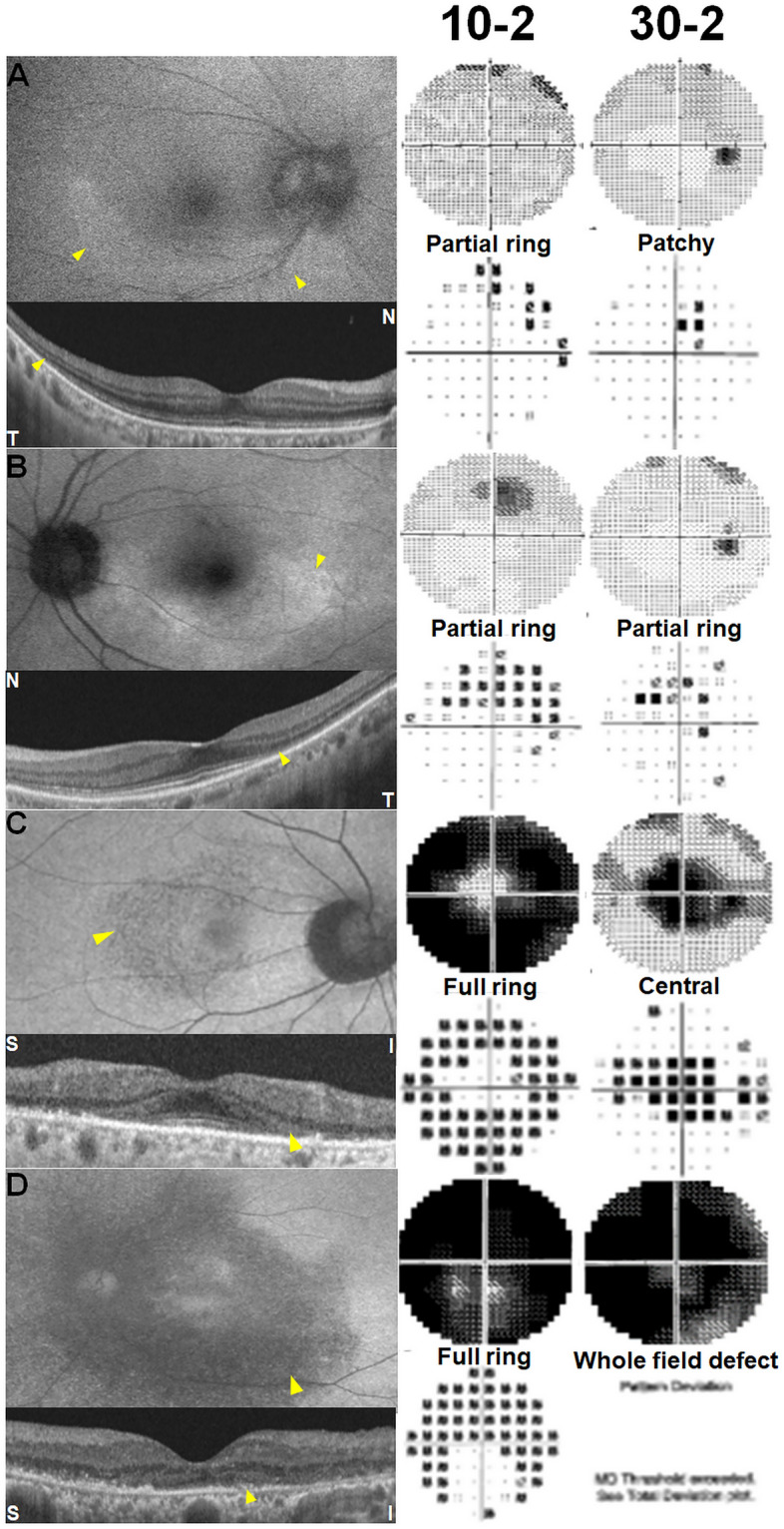
Representative examples of scotoma patterns noted in 10-2 and 30-2 Humphrey visual field (VF) tests noted in 4 patients with hydroxychloroquine retinopathy: patchy scotoma, partial- or full-ring scotoma, ring scotoma, central scotoma, and whole-field defect. In each case, results from fundus autofluorescence (FAF, top left), optical coherence tomography (OCT, bottom left), grayscale map (top right, with text of VF protocol), and pattern deviation map (bottom right) are presented. All patients showed abnormalities, including photoreceptor loss on OCT, and hyper- or hypo-autofluorescence on FAF in the parafoveal or pericentral areas. Yellow arrowheads indicate the areas of retinal damage. N = nasal; T = temporal; S = superior; I = inferior.

We evaluated the characteristics of abnormal findings on VF tests and the diagnostic capabilities of VFs using different strategies. For the interpretation of VF results, abnormal VFs were defined as follows: a total of ≥ 3 points with *P* < 0.05, including ≥ 1 point with *P* < 0.01, on the pattern deviation map^15,16^. In patients with abnormal VFs, the scotoma pattern was determined according to its shape. Specifically, patchy scotoma (consecutive scotoma points with an extent within 90°; Fig. 1A), partial ring scotoma (arc-shaped scotoma with an extent between one and three quadrants; Fig. 1B), full-ring scotoma (ring-shaped scotoma involving all quadrants; Fig. 1C,D), central scotoma (round scotoma involving the four foveal points but not involving the whole field; Fig. 1C), and whole field defects (whole field loss; Fig. 1D). The scotoma location was further analyzed using a pointwise frequency map^17^, which indicated the proportion of eyes showing abnormality (*P* < 0.05) at each test location in the 10-2 and 30-2 tests among those tested with each of the VF strategies using MATLAB software 2021a (MathWorks, Inc., Natick, MA, USA). The map represents test locations by means of a gray scale from white (zero frequency) to black (highest frequency). The probabilities of all of the test points in the left eye images were converted to the right-eye format by matching identical test locations, leading to one probability for each test location regardless of laterality. Two independent investigators, one glaucoma specialist (K.E.K.) and one retina specialist (S.J.A.), both blinded to the patients’ clinical information, reviewed and interpreted all of the VFs and retinal images. In cases of any discrepancies, consensus was reached through discussion.

### Statistical analyses

Descriptive statistics were obtained for the demographic data, hydroxychloroquine dosage details, clinical characteristics, and VF results of the included patients. Continuous values are presented as mean ± standard deviation. Clinical characteristics and details of hydroxychloroquine use were compared using the Fisher’s exact test (for dichotomous variables), student’s *t*-test (for normally distributed continuous variables), or Mann–Whitney test (for continuous variables violating normality assumption assessed by Shapiro–Wilk test). The sensitivity (the number of diseased eyes showing VF abnormality on the VF test [correctly classified] divided by the number of eyes with retinopathy examined using the same strategy) and specificity (the number of normal eyes with normal VF results using the protocol divided by all normal eyes receiving the same strategy) of the 10-2 and 30-2 strategies of the Humphrey VF test were calculated and compared using McNemar’s test for the eyes with results from both tests. Statistical analyses were performed using SPSS software (version 23.0; IBM Corp., Armonk, NY, USA). Statistical significance was set at *P* < 0.05.

## Results

### Patient demographics and clinical characteristics

Among the 1,078 Korean patients included in the present study, 68 (6.3%) were diagnosed with hydroxychloroquine retinopathy. Table 1 summarizes the demographic data and clinical characteristics of patients with hydroxychloroquine retinopathy. The retinopathy group comprised 66 women and two men, and the mean age was 55.2 ± 13.7 years. In that group, the mean duration of hydroxychloroquine use was 16.1 ± 6.7 years. The mean cumulative hydroxychloroquine dose was 1,469.0 ± 645.4 g, and the mean daily dose divided by body weight was 5.0 ± 1.4 mg/kg.

**Table 1:** Demographic data and clinical characteristics of the included patients screened for hydroxychloroquine retinopathy.

Characteristics	Retinopathy (n = 68)
Sex, female (%)	66 (97.1)
Mean age, years (range)	55.2 ± 13.7 (16–81)
Diagnosis, SLE:RA:other* (%)	38:28:2 (55.9:41.2:2.9)
Mean daily dose, mg (range)	253.4 ± 73.8 (131.3–400)
Mean daily dose/body weight, mg/kg (range)	5.0 ± 1.4 (3.1–8.5)
Mean daily dose/ideal body weight^†^, mg/kg (range)	5.0 ± 1.5 (2.9–8.7)
Mean duration of hydroxychloroquine use, yrs (range)	16.1 ± 6.7 (3–33)
Mean cumulative dose, g (range)	1,469.0 ± 645.4 (328.5–3,066)
Mean cumulative dose/body weight, g/kg (range*)*	28.9 ± 14.2 (6.6–68.4)
Mean cumulative dose/ideal body weight, g/kg (range)	29.6 ± 14.0 (6.3–63.5)

*RA* Rheumatoid arthritis, *SLE* Systemic lupus erythematosus.

*Other medical indications included mixed connective tissue disease and Sjögren's syndrome.

^†^Calculated by Devine formula (IBW = 50 + 2.3 kg per inch over 5 feet [for men] or 45.5 + 2.3 kg per inch over 5 feet [for women]).

### VF findings in patients with hydroxychloroquine retinopathy

Diverse patterns of VF defects were found with the 10-2 and 30-2 tests, including partial- or full-ring scotoma, central scotoma, patchy scotoma, and whole-field defects, as shown in Fig. 1. The 10-2 test revealed that partial- or full-ring scotoma was the most common finding (64.3%), followed by patchy (5.2%) and central (4.3%) scotomas (Table 2). Similar patterns of VF defects were found with the 30-2 test, but with different frequencies (Table 3). Partial- or full-ring scotoma was most observed in the test, as noted in approximately 63% of the eyes. In subgroup analyses according to retinopathy pattern, both 10-2 and 30-2 tests showed partial- or full-ring scotoma as the most common VF finding in eyes with pericentral and parafoveal retinopathy.

**Table 2:** Typical patterns of scotoma on the Humphrey 10-2 standard automated perimetry in subgroups according to the pattern among overall patients and those with early retinopathy.

Groups	Partial- or full-ring scotoma	Patchy scotoma	Central scotoma	Whole-field defect
Overall (n = 115)	74 (64.3%)	6 (5.2%)	5 (4.3%)	2 (1.7%)
Pericentral (n = 70)	37 (52.9%)	5 (7.1%)	1 (1.4%)	0
Parafoveal (n = 23)	17 (73.9%)	1 (4.3%)	4 (17.4%)	0
Mixed (n = 22)	20 (90.9%)	0	0	2 (9.1%)
Early retinopathy (n = 37)	12 (32.4%)	3 (8.1%)	0	0
Pericentral (n = 32)	8 (25%)	3 (9.4%)	0	0
Parafoveal (n = 5)	4 (80%)	0	0	0

**Table 3:** Typical patterns of scotoma on the Humphrey 30-2 standard automated perimetry in subgroups according to the pattern among overall patients and those with early retinopathy.

Groups	Partial- or full-ring scotoma	Whole-field defect	Patchy scotoma	Central scotoma
Overall (n = 134)	85 (63.4%)	12 (9.0%)	8 (6.0%)	7 (5.2%)
Pericentral (n = 80)	58 (72.5%)	0	6 (7.5%)	0
Parafoveal (n = 26)	11 (42.3%)	0	2 (7.7%)	7 (26.9%)
Mixed (n = 28)	16 (57.1%)	12 (42.9%)	0	0
Early retinopathy (n = 45)	21 (46.7%)	0	6 (13.3%)	0
Pericentral (n = 38)	17 (44.7%)	0	6 (15.8%)	0
Parafoveal (n = 7)	4 (57.1%)	0	0	0

**Figure 2:**
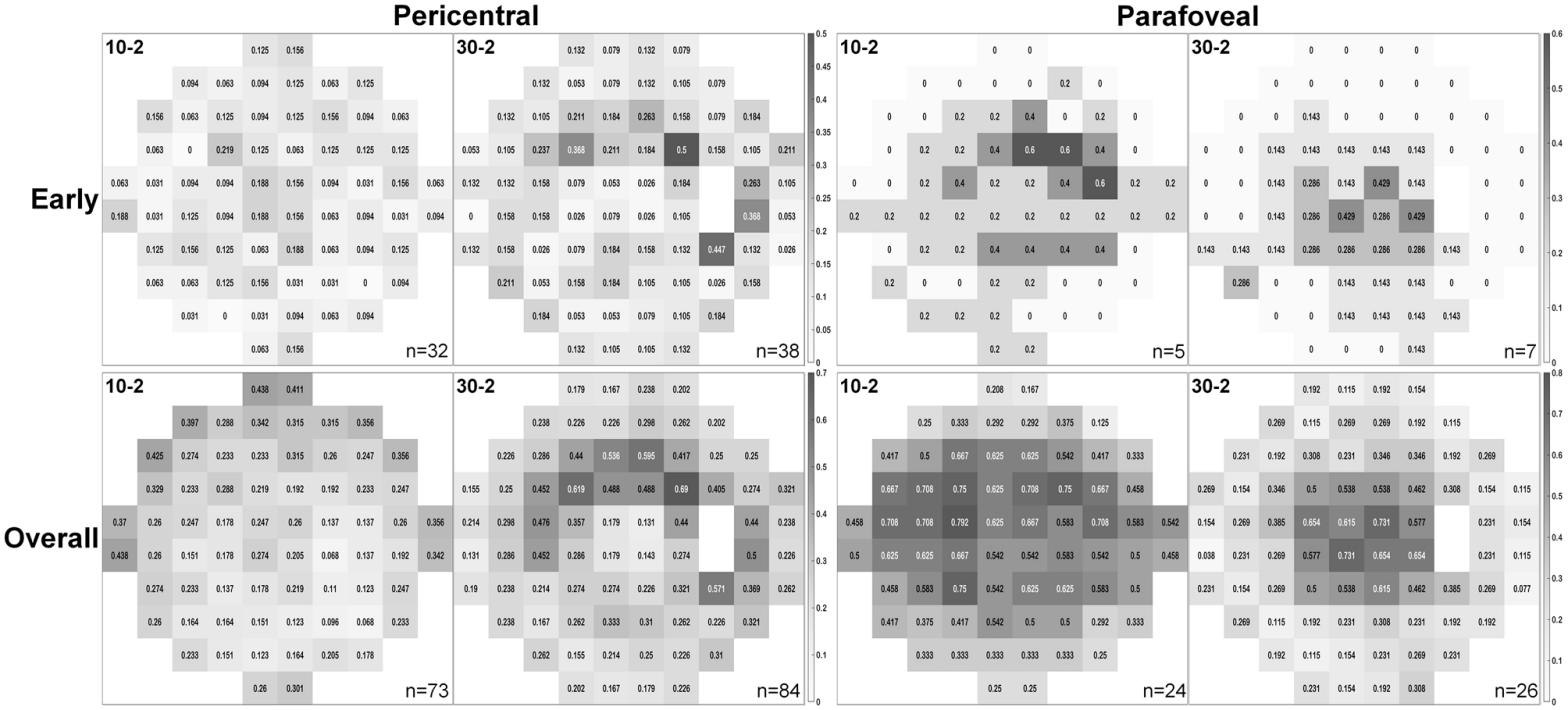
Pointwise frequency map of scotoma for pattern deviation maps of 10-2 and 30-2 tests in early and overall eyes with hydroxychloroquine retinopathy. Probabilities (the proportion of eyes showing abnormality at each test location in the 10-2 and 30-2 tests among those tested with each of the VF strategies) are indicated as numbers within each test point and also represented by using grayscale in each group of parafoveal and pericentral retinopathy among overall and early cases. The numbers in the lower right corner indicate the sample sizes (n) for the frequency maps.

Figure 2 presents pointwise frequency maps based on pattern deviation maps from 10-2 and 30-2 tests of eyes with pericentral and parafoveal hydroxychloroquine retinopathy. In eyes with pericentral retinopathy, the superior peripheral rim in the 10-2 test and the superior arc in the 30-2 test were noted with high frequency, demonstrating spatial concordance between the tests.

### VF findings in early retinopathy

Most early parafoveal cases (80%) demonstrated partial- or full-ring scotoma in the 10-2 test, whereas early pericentral cases showed diverse patterns, such as partial ring and patch scotoma, in either the 10-2 or 30-2 test. In early pericentral retinopathy, 10-2 test results were mostly normal, showing no scotoma. In contrast, the 30-2 test mostly detected scattered scotoma points, which were usually located in the superior area (Supplemental Fig. S2).

In pointwise frequency maps for early pericentral eyes, the 10-2 test generally showed a very low frequency at all test locations. However, the 30-2 test revealed a few points with high frequencies, which were located mostly in the superior field. In early parafoveal eyes, the superior area was more commonly affected than other areas in the 10-2 test, and that area corresponded with more central location in the 30-2 test.

### Diagnostic performance of VF test using different strategies in subgroups of retinopathy patterns and severities

Table 4 presents a diagnostic validity comparison between the 10-2 and 30-2 tests for subgroups categorized by the hydroxychloroquine retinopathy pattern. In overall eyes with parafoveal retinopathy, the 10-2 and 30-2 tests showed sensitivities of 95.2% and 85.7%, respectively, with no significant difference (*P* = 0.480). However, in those with pericentral retinopathy, the 30-2 test (95.7%) showed a significantly higher sensitivity than the 10-2 test (77.1%, *P* < 0.001).

**Table 4:** Diagnostic performance of the Humphrey 10-2 and 30-2 visual field (VF) examinations for hydroxychloroquine retinopathy in subgroups according to retinopathy patterns and severities.

Groups	Sensitivity*			Specificity*		
	10-2 test	30-2 test	*P*	10-2 test	30-2 test	*P*
Parafoveal retinopathy	95.2% (20/21)	85.7% (18/21)	0.480	89.6% (1,105/1,233)	84.8% (1,046/1,233)	< 0.001
Early	80% (4/5)	40% (2/5)	0.480			
Moderate	100% (5/5)	100% (5/5)	1.0			
Severe	100% (11/11)	100% (11/11)	1.0			
Pericentral retinopathy	77.1% (54/70)	95.7% (67/70)	**< 0.001**			
Early	53.1% (17/32)	90.6% (29/32)	**0.002**			
Moderate	95.7% (22/23)	100% (23/23)	1.0			
Severe	100% (15/15)	100% (15/15)	1.0			
Mixed retinopathy	100% (22/22)	100% (22/22)	1.0			
Moderate	100% (2/2)	100% (2/2)	1.0			
Severe	100% (20/20)	100% (20/20)	1.0			

Included were those that received both 10-2 and 30-2 tests (113 eyes with retinopathy and 1,233 healthy eyes).

*Sensitivity = no. of diseased eyes showing VF abnormality on the strategy (correctly classified) / no. of all the eyes with retinopathy examined using the same strategy; specificity = no. of normal eyes with normal VF results on the strategy/ no. of all healthy eyes that received the same strategy.

Significant values are in bold.

A subgroup comparative analysis of diagnostic performance according to disease severity showed better 30-2 test sensitivity in early pericentral retinopathy compared to the 10-2 test (90.6% vs. 53.1%, *P* = 0.002). The 10-2 and 30-2 tests showed a remarkable difference in early parafoveal retinopathy (80.0% and 40.0%, respectively), though it was not statistically significant (*P* = 0.480).

### Distinction of VF abnormalities between different test strategies in two patterns of disease

**Figure 3:**
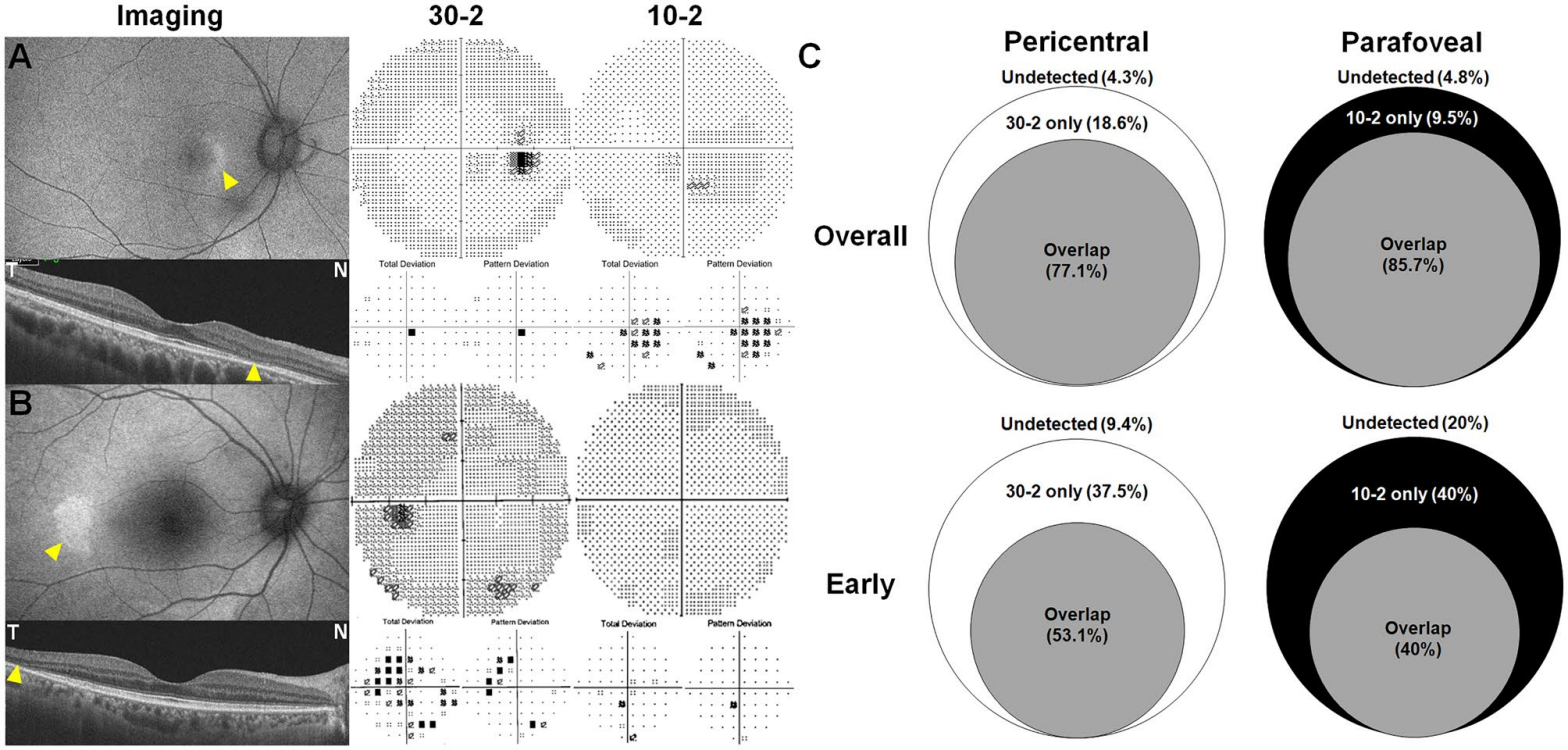
Examples of visual field (VF) results showing disparity in abnormality between 10-2 and 30-2 tests in eyes with (**A**) parafoveal and (**B**) pericentral retinopathy, and (**C**) Venn diagram showing disparity between the tests. (**A**) In the eye with parafoveal retinopathy, the 30-2 test shows an ambiguous finding, whereas the 10-2 test shows a temporal patchy scotoma. (**B**) In the eye with pericentral retinopathy, the 30-2 test shows a partial-ring scotoma, while the 10-2 test plots are essentially normal. The yellow arrowheads indicate abnormal findings on fundus autofluorescence (FAF, top left) and optical coherence tomography (OCT, bottom left) imaging. (**C**) Percentage of eyes classified as having an abnormal VF with the 30-2 (white) and 10-2 (black) tests for each retinopathy pattern among overall and early cases. Gray indicates those with the same results (overlap) between the 10-2 and 30-2 tests.

Among the 113 eyes that underwent both the 10-2 and 30-2 tests for hydroxychloroquine retinopathy screening, 16 (14.2%), including 2 of 21 (9.5%) parafoveal eyes and 14 of 70 (20%) pericentral eyes, revealed distinction of VF abnormalities between different test strategies. Almost all eyes (93.8%) showing the distinction had early retinopathy. Photographic examples of such early cases are presented in Fig. 3. These results reflect different detection capabilities between the two test strategies according to the spatial characteristics of retinal damage. Based on Venn diagrams showing the proportion of patients with abnormal VFs in the 30-2 and 10-2 test results (Fig. 3C), the distinction between different test strategies in the two patterns of disease was remarkable in early retinopathy. Approximately 40% of the eyes with early parafoveal or pericentral retinopathy revealed abnormal VFs in the results from only one test (either 10-2 or 30-2).

## Discussion

The AAO recommends regular ophthalmic screening for patients on long-term hydroxychloroquine therapy^3^. According to the AAO recommendation, VF examination plays a key role in screening, as it provides evidence for subjective and functional abnormalities^3^. Whereas several studies have reported VF findings for parafoveal retinopathy^4,6,18–20^, findings for pericentral retinopathy are relatively unknown, and early detection of retinopathy remains challenging. In this context, the present study reported several characteristic VF patterns for each retinopathy pattern, and also findings from early retinopathy. We believe that our results provide clinical evidence for the most recent AAO guidelines on VF test protocols and also serve as a basis for greater specification on VF examinations for hydroxychloroquine retinopathy screening.

Although severe cases show evident findings in any screening test, VF confirmation of abnormal findings would be clinically useful for the detection of early disease. Accordingly, this study investigated VF findings in overall parafoveal and pericentral retinopathy and separately in early cases. In addition to the characteristic ring scotoma^4,6,19,20^, our study showed diverse patterns of VF defects in a relatively large number of cases with varying severity and retinopathy patterns. These scotoma patterns may originate from different extents of structural defects in hydroxychloroquine retinopathy^14,21^. However, such specific patterns were observed less frequently in early cases, requiring additional consideration for detection. Abnormal findings should be cautiously examined for structural correlation using high-resolution imaging, such as spectral-domain OCT, or for confirmation using other functional tests. Furthermore, scotoma should be reproducible by retest unless there are characteristic (or evident) patterns of scotoma in retinopathy. In addition, repeat tests should be integrated for better interpretation of VF results.

Particularly, early pericentral retinopathy poses clinical challenges for detection in several ways. First, early pericentral damages may not be covered by conventional OCT or FAF imaging or the 10-2 VF test^14,21^. Second, as the 10-2 test consists of more test points within the central 10° than those in paracentral areas of the same size on the 30-2 test (i.e., 68 test points within the central 10° vs. 64 between 10° and 30°), central damage on the 10-2 test can be more easily identified and interpretable as a recognized pattern than a similar degree of paracentral damage on the 30-2 test. This can result in lower sensitivity of the 30-2 test for early pericentral damage relative to that of the 10-2 test for early parafoveal damage. Furthermore, FAF findings in early cases or OCT findings in pericentral areas may not be easily identifiable by screening physicians. Performing both 10-2 and wider tests at one visit may minimize misdetection of pericentral and parafoveal retinopathy; however, this is technically difficult in the actual clinical practice owing to cost, insurance claims, patient fatigue, and time considerations. Thus, screening with one single test would be inevitable, although future development of a combined test by commercial manufacturers, covering both center and periphery, would be a helpful alternative.

In the present study, each of the 10-2 and 30-2 VF test strategies showed variable diagnostic performance and distinction in abnormality according to the retinopathy pattern and severity. The 30-2 test demonstrated a significantly higher diagnostic performance than 10-2 in patients with early pericentral retinopathy. The 10-2 test showed higher sensitivity than 30-2 in eyes with early parafoveal retinopathy, albeit the difference was statistically insignificant. We speculate that this insignificance may be attributable to the small number of patients with early parafoveal retinopathy screened by both tests in our study population (n = 5). By investigating the source of differences in sensitivities between the 10-2 and 30-2 test strategies, we noted that contradictory results arose mostly in cases of early retinopathy. This implies that appropriate selection of the VF test strategy may significantly affect detection ability for early-stage eyes.

Our results provide robust evidence for the most recent AAO guidelines, which have called for wider tests for Asians^3^. In addition, we believe that more detailed suggestions for further VF examinations can be provided based on our results. Given that pericentral disease can also be observed in non-Asian patients, and that parafoveal patterns do arise in Asians (as was observed in the present study)^9^, the retinopathy pattern should be the most important factor determining recommendations for VF strategies. However, there are currently no specific clinical biomarkers, other than ethnic information, for pre-test estimation of retinopathy patterns. Thus, clinicians are advised to select VF test strategies according to patients’ ethnicity. Nonetheless, if confirmation of structural damage by VF examination is necessary for the definite diagnosis of retinopathy for those with ambiguous or normal VF findings, modification of VF strategies based on the specific patterns of retinal damage may be required.

Several limitations should be considered when interpreting our results. First, the subjective nature and high intra-individual variability of the VF test may limit our results on the pattern and prevalence of VF defects in patients taking hydroxychloroquine. To overcome this limitation, we included only patients with reliable VFs, and we confirmed the identified patterns of VF defects at subsequent visits. Second, we used modified diagnostic criteria; as a result, our retinopathy group showed evident structural defects confirmed by high-resolution imaging. However, discrepancies between structural and functional tests have been reported^19,22^. Thus, patients with only structural damage at the time of our analysis may have shown functional damage later on, and vice versa in those with functional damage only. Additionally, lack of a normal control group is another limitation of this study; however, it was very difficult to retrospectively identify a large number of normal controls not taking hydroxychloroquine who received the same tests as the retinopathy group, both 10-2 and 30-2 tests. Moreover, VF results may be influenced by fatigue particularly when successive VF examinations are performed. However, as the two VF strategies were performed randomly in our patients, we believe that there would be no remarkable bias favoring either 10-2 or 30-2 strategy. Finally, the included patients were all Koreans with predominantly pericentral diseases, resulting in only a small number of patients with parafoveal diseases. This requires consideration in interpreting the results obtained from statistical analyses. Thus, further investigation in populations of diverse ethnicities is needed to finalize the data on VF and the diagnostic performance of different VF testing strategies in patients with hydroxychloroquine retinopathy.

In conclusion, our study revealed a higher sensitivity of the 30-2 VF test for detecting hydroxychloroquine retinopathy relative to the 10-2 test in patients with pericentral retinopathy. The distinction between the 10-2 and 30-2 strategies in the two patterns of disease was noted mostly in early retinopathy cases, which suggests that test strategies should be judiciously selected to effectively cover the area of structural damage for early detection of retinopathy. Our results may serve to guide the appropriate selection of VF strategy according to the pattern of retinopathy for screening of hydroxychloroquine retinopathy.

## Supplementary Information

Supplementary Information 1.

Supplementary Information 2.

Supplementary Information 3.
