# Supplementary material for: Visual field examinations using different strategies in Asian patients taking hydroxychloroquine
Source: Sci Rep. 2022 Aug 30;12:14778. doi: 10.1038/s41598-022-19048-0 (PMC9427842; doi:10.1038/s41598-022-19048-0)
Supplement: Supplementary file 1 — Supplementary Information 1. [file 41598_2022_19048_MOESM1_ESM.docx]

**Supplemental Figure Legends**

**Supplemental Figure S1.** Flowchart of inclusion and exclusion criteria of present study and numbers of participants meeting criteria. The red text indicates the analyses performed for the population on the left. VF = visual field.

**Supplemental Figure S2**. **Photographic examples of visual field (VF) findings in eyes with early pericentral retinopathy.** Horizontal and vertical scans of optical coherence tomography demonstrate focal, pericentral photoreceptor damage (yellow arrowheads). Pattern deviation maps on 30-2 VF tests show nonpatterned, variable scotoma points; however, the points are commonly located in the paracentral area. I = inferior; N = nasal; PD = pattern deviation; S = superior; T = temporal.
