# Supplementary figures and images for: Visual field examinations using different strategies in Asian patients taking hydroxychloroquine
Source: Sci Rep. 2022 Aug 30;12:14778. doi: 10.1038/s41598-022-19048-0 (PMC9427842; doi:10.1038/s41598-022-19048-0)

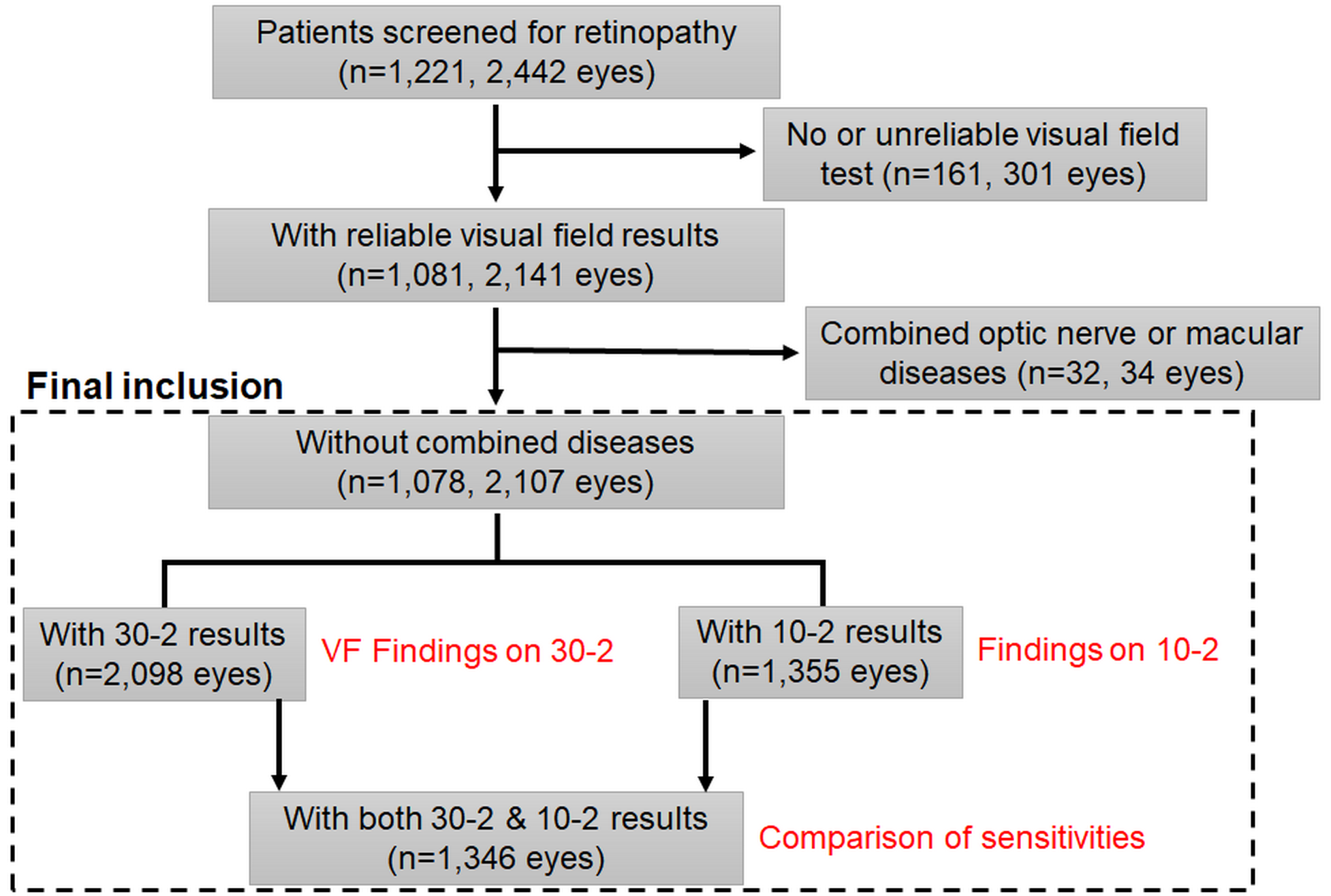

Supplement: Supplementary file 2 — Supplementary Information 2. [file 41598_2022_19048_MOESM2_ESM.tif]

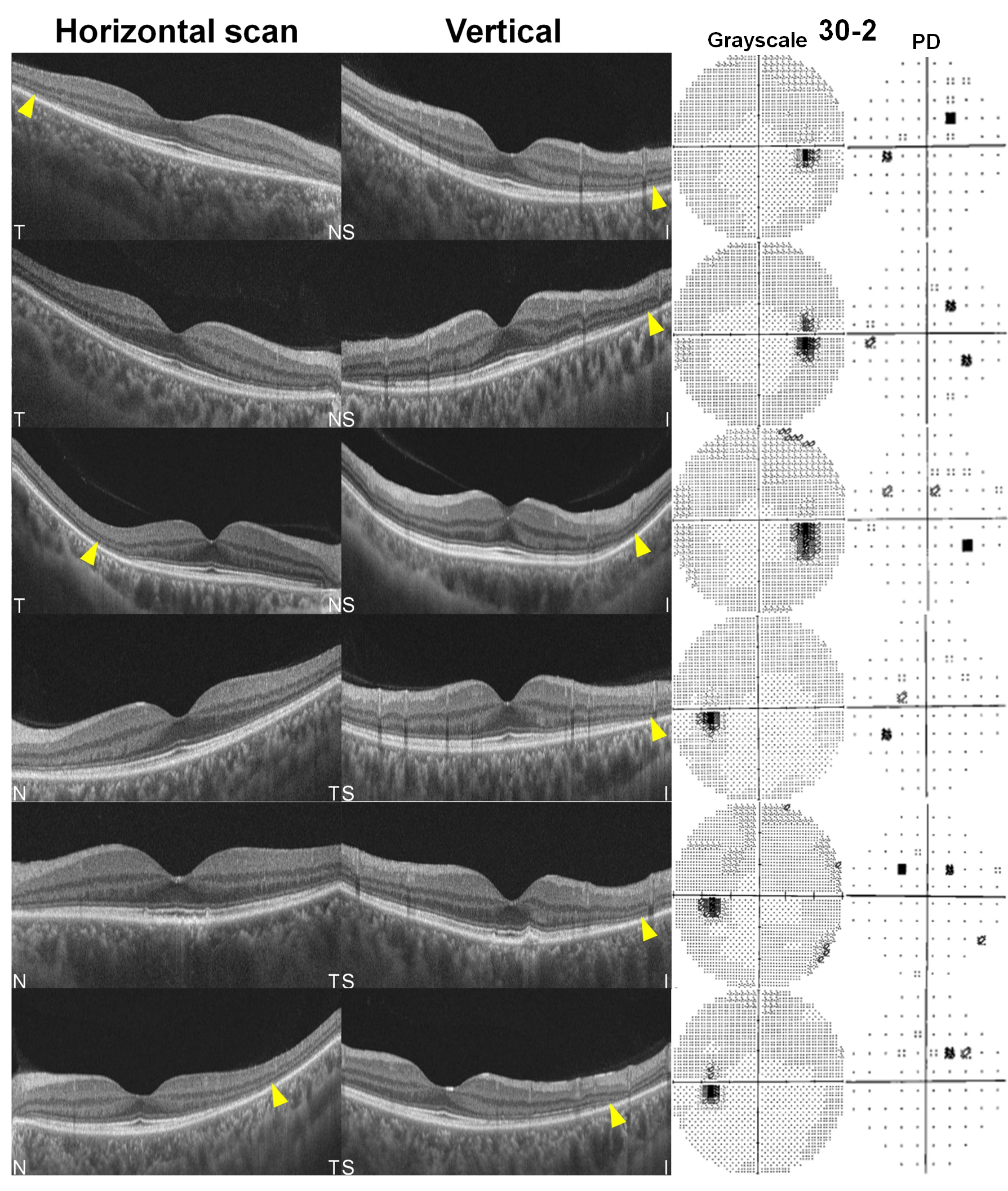

Supplement: Supplementary file 3 — Supplementary Information 3. [file 41598_2022_19048_MOESM3_ESM.tif]
